# Impact of tele-antimicrobial stewardship at two small community hospitals in partnership with an academic medical center: two years of experience

**DOI:** 10.1017/ash.2024.418

**Published:** 2024-09-23

**Authors:** Jennifer K. Ross, Aditya A. Chandorkar

**Affiliations:** 1 Fairview Pharmacy Services, Minneapolis, MN, USA; 2 M Health Fairview University of Minnesota Medical Center, Minneapolis, MN, USA; 3 Division of Infectious Diseases and International Medicine, University of Minnesota, Minneapolis, MN, USA

## Abstract

**Objective:**

To analyze the impact of a fully remote tele-antimicrobial stewardship program (TASP) at two small community hospitals in partnership with an academic medical center.

**Design:**

Retrospective survey from August 1, 2020, to July 31, 2022.

**Setting:**

A TASP, co-led by an infectious diseases (ID) physician and ID pharmacist, was implemented at M Health Fairview (MHF) Northland, a 21-bed hospital, and at MHF Lakes, a 49-bed hospital. The ID physician and ID pharmacist are located at the University of Minnesota Medical Center.

**Methods:**

Antimicrobial stewardship interventions were tracked monthly. Restricted antimicrobial days of therapy per 1000 patient days (DOT/1000 PD) were also tracked monthly and two years pre and postimplementation data were compared. All annual antimicrobial expenditures were followed.

**Results:**

For the first two TASP years, a total of 789 antimicrobial interventions were made with 85.6% being accepted. Restricted antimicrobial use trended down from 142.93 to 113.97 DOT/1000 PD at MHF Northland. A smaller decrease from 106.31 to 103.12 DOT/1000 PD was seen at MHF Lakes. Annual antimicrobial costs per total patient days decreased. MHF Northland hospital’s annual antimicrobial expenditures per total patient days fell from $18.89 in 2019 (preimplementation) to $6.64. MHF Lakes followed a similar trend, decreasing from $11.20 to $5.36.

**Conclusions:**

A fully remote TASP in partnership with an academic medical center for two small community hospitals resulted in high rates of accepted interventions sustained over two years. Restricted antimicrobial use and antimicrobial costs trended down.

## Introduction

Small hospitals make up a significant portion of hospitals across the United States.^1,2^ In 2021, 75% (n = 3869) of U.S. hospitals had less than 200 beds. Antimicrobial use, *Clostridioides difficile* infection, and drug-resistant bacteria rates in smaller hospitals have been shown to be similar when compared to larger institutions.^3–5^ These sites often lack infectious diseases (ID) specialists.^6^ In 2017, Medicare data showed nearly 80% of counties, correlating to an estimated 208 million people in the United States, had low or no ID provider coverage.^7^ Antimicrobial stewardship programs (ASPs) have proven to be effective in improving patient outcomes, optimizing antimicrobial use, and decreasing costs.^6,8^ Many small hospitals are less likely to have a formalized ASP and meet all core elements set forth by the Centers for Disease Control and Prevention (CDC).^8,9^ Adequate staffing, information technology resources, and financial support are proven barriers to successful ASP implementation and sustainability at small hospitals.^1,6^

Published experiences on ASP implementation stem from larger hospitals with many being academic medical centers.^1^ The Society for Healthcare Epidemiology of America (SHEA)/Infectious Diseases Society of America (IDSA) antimicrobial stewardship guidelines recommend ASPs be led by an ID provider with advanced stewardship training or co-led by an ID provider and a clinical pharmacist with advanced ID training.^10,11^

Tele-antimicrobial stewardship programs (TASPs) have emerged as a creative alternative to meet regulatory ASP requirements and optimize antimicrobial use at small hospitals. Three TASPs models have been described in the literature, namely fully remote, integrated, and collaborative.^6^ Fully remote TASPs include an off-site ID specialist who conducts prospective audit with intervention and feedback (PAIF) on patient cases and interacts with on-site prescribers through various telehealth communication systems. In fully remote models, on-site pharmacist involvement is minimal. In contrast, integrated TASP models bring together remote ID specialists and on-site providers at least once weekly to review patients, with most of the day-to-day ASP activities being led by on-site pharmacists. A collaborative TASP focuses on building antimicrobial stewardship education and resources longitudinally with the on-site pharmacist leading all the daily ASP work.^6^ ID specialists who lead TASPs may be found within a health system to support smaller hospitals or acquired from a non-affiliated private practice, hospital, or private telehealth entity to provide TASP services.^6^

M Health Fairview – University of Minnesota Medical Center (UMMC) has had a longstanding ASP. UMMC’s ASP utilizes PAIF as the primary antimicrobial stewardship strategy. The success of the ASP at the academic medical center, strong relationships between ID providers and ID pharmacists, and a need to meet regulatory antimicrobial stewardship requirements prompted expansion to two rural, community hospitals within the M Health Fairview (MHF) system. In August 2020, TASPs were implemented at MHF Lakes Hospital in Wyoming, Minnesota, and MHF Northland Hospital in Princeton, Minnesota. Our TASP design is fully remote with elements of integration with on-site providers and pharmacists. To our knowledge, this is the first fully remote TASP with routine communication with on-site pharmacists as part of daily workflows. We describe our TASP experience and outcomes in the first two years.

## Methods

### Central ASP at UMMC

UMMC is an 844-bed, tertiary-care academic medical center within the MHF health system. The ASP was formed in 2007, co-led by an ID provider and ID pharmacist. The UMMC ASP operates five days per week with on-call services in the evenings and weekends. Patient chart review for UMMC is completed by 1.5 FTE ID pharmacists for adult patients on at least one restricted antimicrobial in the electronic health record (Epic Systems Corporation, Verona, Wisconsin). Patients warranting further intervention are reviewed more in-depth during daily rounds with an attending ID provider. A progress note is placed in the patient’s chart and communication with primary providers occurs following rounds. UMMC’s ASP reports to the MHF System Antimicrobial Stewardship Committee and System Pharmacy and Therapeutics Committee, both overseen by MHF Quality and Safety leadership.

### Initiation of TASP at two MHF community hospitals

In preparation for TASP, 0.5 FTE and 0.1 FTE for ID pharmacist and ID provider support, respectively, were approved in 2019. The ID expertise for TASP is based at UMMC. MHF has ten acute care hospitals spanning across Minnesota with the majority (n = 6) located within the Minneapolis-St. Paul metro area. All hospitals within the metro have infectious diseases consult services. On-site ID expertise is not available at MHF Lakes and MHF Northland, two north region hospitals. MHF Lakes is a 49-bed hospital and MHF Northland is a 21-bed hospital. Both offer medical and surgical services and have ICU-level beds with tele-ICU assistance for on-site hospitalists. Emergency medicine, general surgery, obstetrics and gynecology, orthopedics, podiatry, and urology services are also available.

Infrastructure was in place to follow the ASP at UMMC. Many microbiological tests obtained at MHF Lakes and MHF Northland were sent to a centralized microbiology lab at UMMC. Restricted antimicrobial lists in Epic and antimicrobial stewardship note templates had been built prior to TASP initiation. On-site pharmacist coverage occurred on a rotating basis. An ID provider curbside option was also in place, whereby an ID provider at UMMC staffed the ID curbside line pager daily to answer any questions posed by TASP sites. The TASP had the option to recommend ID curbside line if direct ID provider to hospitalist communication was needed. Relationship building with TASP sites started with visits to each hospital to describe the program and workflows with the medical director, lead hospitalists, pharmacists, and infection preventionist.

**Figure 1.**
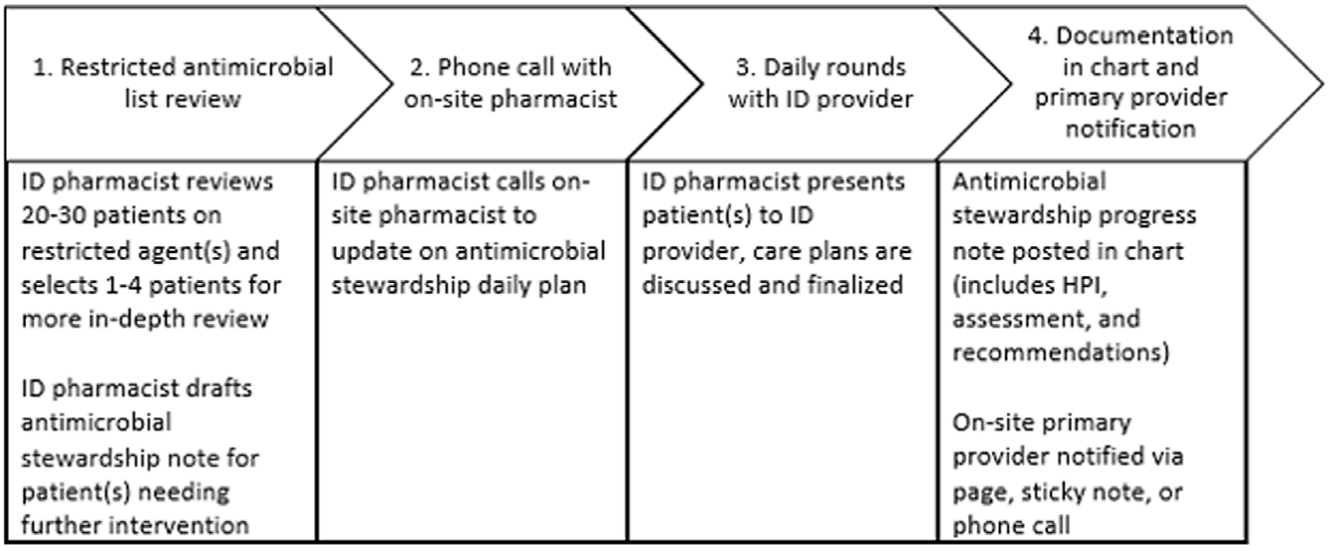
The daily TASP workflow with MHF Lakes Hospital and MHF Northland. ID expertise located at UMMC.

The daily TASP workflow is described in Figure 1. A patient prescribed a restricted antimicrobial populates a real-time list in Epic. The ID pharmacist reviews patients on restricted antimicrobial(s) at each site daily, Monday through Friday. Table 1 outlines restricted antimicrobials. Many internal infectious disease state and drug guidelines exist to aid the ID pharmacist and ID provider in determining antimicrobial stewardship interventions. After patient review, the ID pharmacist calls the on-site pharmacist to share which patient(s) are being reviewed at TASP rounds with the ID provider, discuss other interventions (eg, ordering MRSA nares PCR test), and to see if there are any additional questions or concerns from each TASP site. The ID pharmacist drafts a progress note on patient(s) warranting further intervention, then rounds with the ID provider to finalize recommendations. An antimicrobial stewardship team progress note is placed in the chart and the ID pharmacist reaches out to on-site hospitalists. If TASP interventions are not accepted, the ID pharmacist clarifies rationale with site hospitalist or pharmacist first, may elect to re-round with ID provider or escalate to ID provider for direct communication with on-site primary provider.

**Table 1.** Restricted antimicrobial agents at MHF TASP hospitals. Patients prescribed at least one restricted antimicrobial are included in the purview of the TASP

Restricted Antimicrobials
Amikacin
Amphotericin B (conventional and liposomal)
Aztreonam
Bezlotoxumab
Ceftazidime
Ceftazidime/avibactam
Ceftaroline
Ceftolozane/tazobactam
Cefepime
Cefiderocol
Dalbavancin
Daptomycin
Ertapenem
Fidaxomicin
Fosfomycin
Gentamicin
Imipenem/cilastatin
Itraconazole
Linezolid
Meropenem
Micafungin
Piperacillin/tazobactam
Posaconazole
Remdesivir
Tigecycline
Tobramycin
Vancomycin
Voriconazole

### Data collection and statistics

A report was generated that included all patients reviewed by the ID pharmacist at each site. This report helped to identify patients where TASP had formal interventions and placed a progress note. TASP accepted intervention rates were manually reviewed monthly. Interventions recommended by TASP that were not acted on within 24 hours of progress note placement were counted as rejected. Patients were counted multiple times if more than one TASP progress note was placed. We did not collect data on intervention type, such as antimicrobial de-escalation, duration of therapy, switch from intravenous to oral therapy, and tests to aid workup, given the manual nature of this data collection and inability to include intervention specifics in generated report.

Restricted antimicrobial drug use was measured for each TASP hospital defined by days of therapy per 1000 patient-days (DOT/1000 PD). We elected not to include nonrestricted agents because those agents fell outside the current realm of TASP purview. These data are readily available in Epic via the Bugsy module and tracked monthly. Mean DOT/1000 PD averages two years pre and postimplementation data were compared. All annual antimicrobial costs were calculated on a per patient day basis. This study was reviewed by the University of Minnesota Institutional Review Board and deemed nonhuman research.

## Results

### TASP intervention acceptance rates

A total of 2,249 patients were reviewed by the ID pharmacist during the study period: 852 patients at MHF Northland and 1,397 patients at MHF Lakes. Totally, 789 interventions were made between August 2020 and July 2022: 299 interventions at MHF Northland and 490 interventions at MHF Lakes as shown in Table 2. The average mean accepted intervention rates were similar between the two hospitals, 85% at MHF Northland and 86% at MHF Lakes. Notably, only 55% TASP interventions were accepted initially at MHF Lakes. The accepted intervention rate rose to 72% in the next month, mirroring the rate at MHF Northland. In November 2020, both sites’ accepted intervention rate was above 80%, which was largely maintained thereafter.

**Table 2.** Monthly TASP accepted and total interventions

Month	MHF Northland Hospital Accepted/Actual Interventions (%)	MHF Lakes Hospital Accepted/Actual Interventions (%)
Aug 2020	6/7 (85%)	6/11 (55%)
Sep 2020	12/17 (71%)	18/25 (72%)
Oct 2020	8/11 (72%)	21/25 (82%)
Nov 2020	11/13 (85%)	31/39 (82%)
Dec 2020	7/8 (88%)	28/33 (85%)
Jan 2021	12/14 (86%)	15/17 (88%)
Feb 2021	12/15 (80%)	15/18 (83%)
Mar 2021	7/8 (88%)	19/23 (83%)
Apr 2021	11/13 (85%)	22/24 (92%)
May 2021	9/10 (90%)	20/21 (95%)
Jun 2021	11/13 (85%)	15/16 (94%)
Jul 2021	9/12 (75%)	15/18 (83%)
Aug 2021	12/15 (80%)	23/26 (88%)
Sep 2021	15/16 (94%)	16/19 (84%)
Oct 2021	19/20 (95%)	15/17 (88%)
Nov 2021	13/13 (100%)	11/13 (85%)
Dec 2021	11/12 (92%)	18/19 (95%)
Jan 2022	8/10 (80%)	16/17 (94%)
Feb 2022	13/13 (100%)	14/15 (93%)
Mar 2022	10/12 (83%)	21/23 (91%)
Apr 2022	8/11 (72%)	18/21 (86%)
May 2022	11/14 (79%)	12/14 (86%)
Jun 2022	15/16 (94%)	15/17 (88%)
Jul 2022	5/6 (83%)	17/19 (89%)
*Totals*	*255/299 (85%)*	*421/490 (86%)*

### Restricted antimicrobial use and expenditures

**Figure 2.**
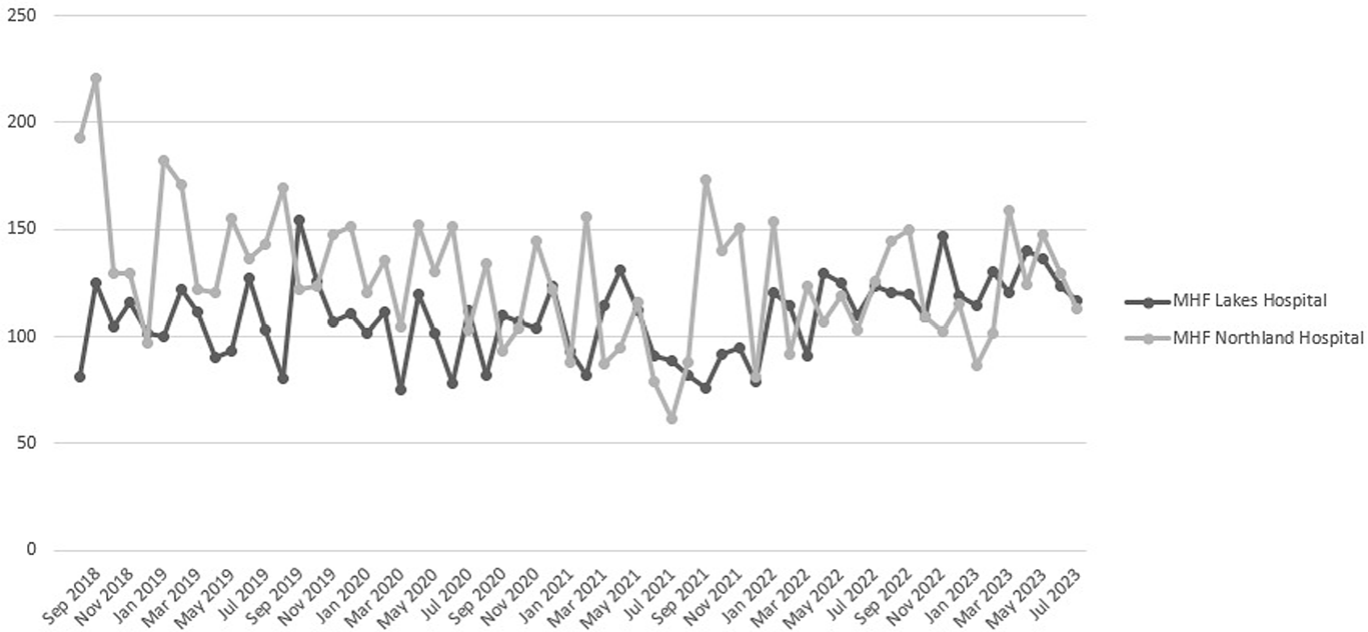
Monthly restricted antimicrobial use (DOT/1000 PD).

Restricted antimicrobial utilization during the intervention period was 103.12 DOT/1000 PD and 113.97 DOT/1000 PD at MHF Lakes and MHF Northland, respectively, compared to 106.31 DOT/1000 PD and 142.13 DOT/1000 PD during the preintervention period. MHF Lakes experienced a small reduction of 3% in restricted antimicrobial use whereas MHF Northland experienced a larger reduction of 19.8%. Monthly restricted antimicrobial utilization for the 24-month baseline and 24-month intervention periods is shown in Figure 2 and Table 3.

**Table 3.** Average restricted antimicrobial utilization for the 24-month baseline and 24-month intervention periods

TASP Hospital	PreTASP (7/2018-7/2020) Average Restricted Antimicrobial DOT/1000 PD	PostTASP (8/2020-7/2022) Average Restricted Antimicrobial DOT/1000 PD
MHF Northland Hospital	141.97	113.97
MHF Lakes Hospital	106.26	103.12

**Figure 3.**
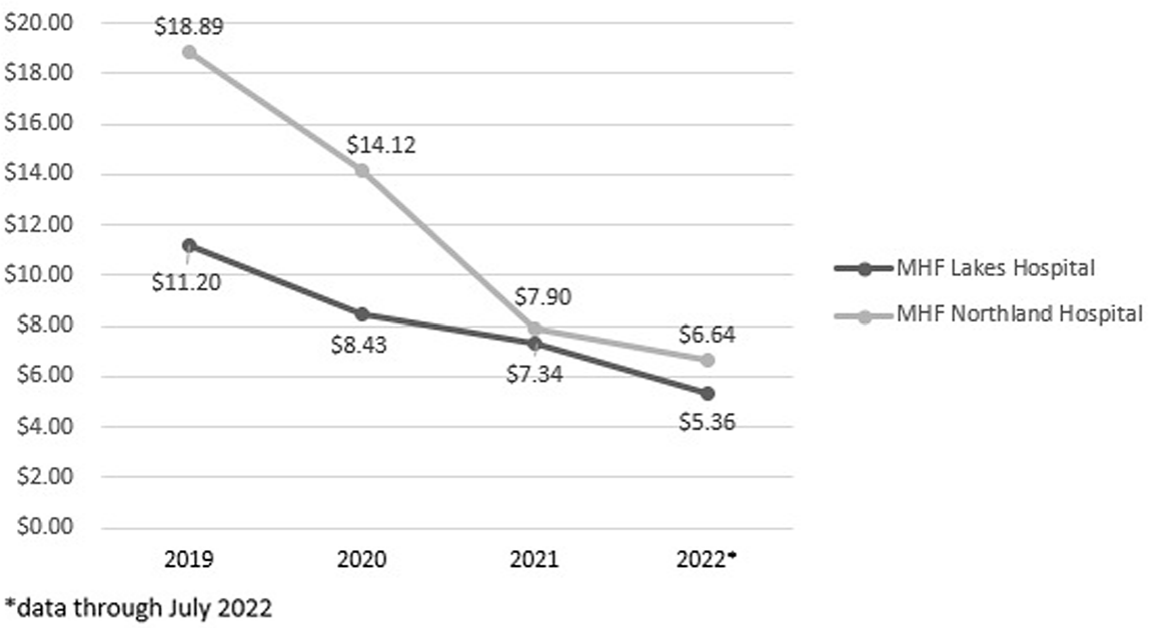
Annual TASP all antimicrobial expenditures per patient day.

MHF Lakes and MHF Northland’s total antimicrobial expenditures for all antimicrobial agents were $95,796 and $85,224, respectively, in 2019 (preTASP implementation). MHF Lakes had 8,556 total patient days, yielding total antimicrobial costs per patient day of $11.20. MHF Northland had 4,512 total patient days. This resulted in total antimicrobial costs per patient day of $18.89. Following TASP implementation, a steady decrease in antimicrobial expenditures was seen. For the year 2022 through July, MHF Lakes antimicrobial costs per patient day were $5.36 ($34,040/6,347 patient days) and MHF Northland were at $6.64 ($20,287/3,054 patient days). Figure 3 shows the annual costs per patient day for each site. This resulted in a 52% reduction at MHF Lakes and a 64.8% reduction at MHF Northland.

## Discussion

This study describes the implementation of an inpatient TASP at two small community hospitals in partnership with an academic medical center within the same health system. Our TASP model may assist small hospitals or health systems in developing successful and sustainable TASPs that meet antimicrobial stewardship regulatory requirements.

During the TASP’s first two years of experience, the acceptance rate of TASP interventions was 85.6% overall. This rate parallels what has been described in other studies at community hospitals with similar TASP models.^12–14^ The on-site education of providers and pharmacists on the TASP structure and workflows enhanced program integration. While the accepted TASP intervention rates were low at first, ranging from 55% to 72% in the first few months, an increase and subsequent sustainment was seen. On-site pharmacists helped further establish trusting relationships between the ID pharmacist and on-site providers. Driven by PAIF, the TASP operated Monday through Friday, ensuring consistency. Previously published data support successes with this core strategy.^15,16^

Our TASP hospitals experienced a reduction in restricted antimicrobial use when compared to baseline. The magnitude in reduction was different between the two hospitals, decreasing 3% at one site compared to a near 20% reduction at the other hospital. MHF Northland had higher baseline restricted antimicrobial use when compared to MHF Lakes, lending to greater opportunities for reduction in restricted antimicrobial use. Improvements in broad-spectrum antimicrobial use have been reported in prior studies, acknowledging heterogeneity in the antimicrobial(s) within the TASP’s scope.^12,13,17–21^ Large decreases of 52% and 64.8% in all antimicrobial costs per patient days despite experiencing higher patient volumes were seen at our TASP sites. Notably, our TASP does not review patients in the emergency department or perioperative surgical antibiotic prophylaxis.

There are several limitations to our data. First, our study is representative of a single health system experience. We did not capture verbal recommendations that occurred during daily correspondence with on-site pharmacists. These recommendations were not recorded with a majority falling within the pharmacist’s scope of practice. Second, we focused on restricted antimicrobial utilization only, creating an opportunity for further investigation of non-restricted antimicrobial use. We focused on antimicrobial use data and did not capture antimicrobial appropriateness or outcomes.

Our TASP was implemented during the COVID-19 pandemic in which our TASP sites took care of COVID-19 patients. The impact of the pandemic on antimicrobial use data trends is not well understood. In addition, our TASPs have periodic reliance on hospitalists with short-term contracts, lending to more difficulties in educating providers about TASP workflows and institutional changes in practice. We aimed to investigate the impact of antimicrobial use on other infections, such as *Clostridioides difficile*, but small hospital sizes combined with low *C.difficile* incidence did not allow for more formal analysis.

The evidence supporting successful and sustainable ASPs at smaller hospitals remains limited. Our fully remote TASP structure with built-in elements of routine collaboration with on-site providers and pharmacists led to consistent communication and trusting relationships. We have worked to continue to develop strong relationships with at least annual visits to each hospital. Leveraging ID expertise and antimicrobial stewardship infrastructure affiliated with an academic medical center eased implementation and sustainability.

In conclusion, we describe a unique TASP model that may be implemented within health systems with a larger, tertiary hospital and surrounding community-based sites. Our data show that TASP interventions can be made through more intense prospective audit and feedback and accepted at high rate at community hospitals with correlating reductions in broad spectrum antimicrobial use and expenditures.
